# Supplementary material for: Toxoplasma gondii Genetic Diversity in Mediterranean Dolphins
Source: Pathogens. 2022 Aug 12;11(8):909. doi: 10.3390/pathogens11080909 (PMC9416038; doi:10.3390/pathogens11080909)
Supplement: Supplementary file 1 [file pathogens-11-00909-s001.zip › pathogens-1839377-supplementary.pdf]

pathogens

MDPI

Supplementary Materials Table S1. Anamnestic and stranding data of the sixteen dolphins selected in the present study.

Case# (ID)	Species	Decomposition Conservation Code	Nutritional Status	Sex	Age	Stranding Date	Stranding Location	Reference
#1 (547/15)	Striped dolphin	2	Moderate	Female	Adult	06/01/2015	Bordighera (Imperia)	This study
#2 (3908/15)	Striped dolphin	2	Good	Female	Adult	16/01/2015	Imperia (Imperia)	[51]
#3 (40548/15)	Striped dolphin	2	Good	Male	New-born-calf	03/05/2015	Imperia (Imperia)	This study
#4 (1267/15)	Striped dolphin	2	Moderate	Female	Adult	08/01/2015	Finale Ligure (Savona)	[14]; this study
#5 (14879/16)	Striped dolphin	3	Poor	Male	Juvenile-subadult	17/02/2016	Bordighera (Imperia)	This study
#6 (16769/17)	Striped dolphin	2	Moderate	Male	Juvenile-subadult	16/02/2017	Savona (Savona)	[5,9,52]; this study
#7 (78983/17)	Striped dolphin	2	Poor	Female	Adult	14/09/2017	Savona (Savona)	[5,14,51]; this study
C#8 (50099/18)	Striped dolphin	2	Good	Female	Juvenile-subadult	02/06/2018	Imperia (Imperia)	This study
#9 (62728/18)	Striped dolphin	2	Poor	Male	Adult	26/06/2018	Bivona (Vibo Valentia)	[5]; this study
#10 (92929/18)	Striped dolphin	3	Good	Male	Juvenile-subadult	30/10/2018	Ispani (Salerno)	This study
#11 (95661/19)	Striped dolphin	2	Poor	Male	Adult	26/10/2019	Belvedere Marittimo (Cosenza)	This study
#12 (24676/20)	Striped dolphin	3	Good	Female	Juvenile-subadult	26/02/2020	Villa San Giovanni (Reggio Calabria)	This study
#13 (38325/20)	Striped dolphin	2	Moderate	Male	Juvenile-subadult	08/05/2020	Corigliano-Rossano (Cosenza)	This study
#14 (51352/20)	Bottlenose dolphin	2	Good	Male	Adult	13/07/2020	Camogli (Genova)	This study
#15 (2564/21)	Striped dolphin	4	Good	Male	Adult	10/12/2020	Laureana Cilento (Sa-lerno)	This study
#16 (24621/21)	Striped dolphin	2	Good	Male	Adult	11/02/2021	Bacoli (Napoli)	This study
